# Left cardiac sympathetic denervation for the treatment of inherited arrhythmia syndromes: salvation for the desperate?

**DOI:** 10.1007/s12471-014-0542-z

**Published:** 2014-03-12

**Authors:** M. Voskuil, J. F. van der Heijden

**Affiliations:** Department of Cardiology, University Medical Center Utrecht, Heidelberglaan 100, 3584 CX Utrecht, the Netherlands

In the large majority of patients, ventricular arrhythmias are caused by myocardial ischaemia and/or infarction because of underlying coronary artery disease. Also, nonischaemic cardiomyopathy, infiltrative disease (e.g. sarcoidosis, amyloidosis) and infectious disease (e.g. viral myocarditis) are reasonably frequent causes of ventricular arrhythmias. Obviously, in these cases, treating the underlying disease is the primary goal. However, ventricular arrhythmias also occur in patients with primary inherited arrhythmia syndromes such as catecholaminergic polymorphic ventricular tachycardia (CPVT) and long-QT syndrome (LQTS). CPVT and LQTS are rare causes of ventricular arrhythmias, and predominantly occur in young patients, with apparently structurally normal hearts. The ventricular arrhythmias occur mainly under conditions of increased sympathetic activity (e.g. physical or emotional stress) [1]. In these patients, besides removal of triggers, the cornerstone of treatment of these diseases is β-adrenoreceptor blockade. Treatment with beta-blockers is currently a class I indication in clinically diagnosed patients suffering from ventricular arrhythmias [2]. Beta-blockade should be titrated up to an effective level. If not sufficient, adding flecainide in the case of CPVT appears to be a sensible approach [3]. Implantation of an internal cardioverter defibrillator (ICD) can be considered in the (rare) patient not adequately controlled with medical interventions. However, the use of an ICD alone is also not the panacea because of the catecholamine release, triggered by the pain and fear generated by ICD shocks, which may subsequently initiate arrhythmic storms and/or multiple shocks and may even lead to death [4]. Therefore, continuation of medical therapy in the maximal tolerable dose is mandatory, also after ICD implantation.

Left cardiac sympathetic denervation (LCSD) may be considered for patients with CPVT and LQTS who suffer from recurrent ventricular tachycardias despite maximal pharmacological therapy [5]. Historically, sympathectomy has been used as a treatment modality for angina pectoris [6]. From the 1970s cardiac sympathetic denervation has been described for the treatment of (treatment-resistant) ventricular arrhythmias [7].

In the current issue of the Netherlands Heart Journal, Olde Nordkamp and co-workers present their experience of LCSD in treating therapy-resistant patients with inherited arrhythmia syndromes. [8] They are a tertiary referral centre for this patient cohort in the Netherlands, being the only centre in this country performing this surgical procedure. The baseline characteristics of the described patients show the disease state, since all patents were on beta-blockade and the majority of patients suffering from CPVT were also taking flecainide. Despite maximal medical therapy these patients were still experiencing adverse events (arrhythmias, syncope). The majority of patients underwent a video-assisted thoracoscopic approach, some using the supraclavicular approach and one patient underwent LCSD via thoracotomy.

The clinical results presented are reassuring; in general, a nice 87 % of symptomatic patients experienced a decreased cardiac event rate after LCSD. However, only 47 % of the patients did not experience any cardiac events, while 53 % of the patients remained symptomatic (although to a lesser extent) during a median follow-up of 34 (IQR 16–77) months. These numbers are comparable with previous reports from other (foreign) centres [9].

This reduction of ventricular arrhythmias after LCSD, however, comes at a cost; the risk for serious peri-procedural complications was 12 % (2 out of 17 patients) in the current patient cohort. One patient had a permanent postoperative Harlequin face and one (severely affected) LQT (type 8) patient died postoperatively because of multi-organ failure after several problems following multiple arrhythmic storms. Furthermore, three patients suffered from a post-procedural pneumothorax and one patient experienced a transient Horner’s syndrome. It is, however, to be expected that more experience with this treatment modality will result in a reduction of the (serious) adverse events in the future.

Therefore, the conclusion of this retrospective analysis investigating the safety and efficacy of LCSD of patients with LQTS and CPVT, not responding well to pharmacological therapy, must be that it offers a valuable addition to the medical therapy for patients with CPVT and LQTS, suffering from repetitive ICD shocks and/or ventricular arrhythmias. However, the other side of the coin must be taken into account in all cases, since it is not a minor intervention without risks. If risks and benefits are discussed and understood properly by the (in general young) patient it offers a potential escape for the often-traumatic events of otherwise untreatable ventricular arrhythmias and ICD shocks.

New, less invasive techniques to decrease sympathetic activity, such as for example a percutaneous modality, which makes use of a steerable radiofrequency catheter by creating lesions to perivascular aligned nerve branches of the renal arteries, might be another option to decrease the adrenergic stress on the heart, thereby decreasing the arrhythmia burden in these patients [10]. The possibilities and potential benefits in the case of this specific indication are, however, purely speculative.

## References

[1] Leenhardt A, Lucet V, Denjoy I (1995). Catecholaminergic polymorphic ventricular tachycardia in children. A 7-year follow-up of 21 patients. Circulation.

[2] Zipes DP, Camm AJ, Borggrefe M (2006). ACC/AHA/ESC 2006 guidelines for management of patients with ventricular arrhythmias and the prevention of sudden cardiac death: a report of the american college of cardiology/American heart association task force and the european society of cardiology committee for practice guidelines (writing committee to develop guidelines for management of patients with ventricular arrhythmias and the prevention of sudden cardiac death). Europace.

[3] Watanabe H, Chopra N, Laver D (2009). Flecainide prevents catecholaminergic polymorphic ventricular tachycardia in mice and humans. Nat Med.

[4] Wolf MJ, Zeltser IJ, Salerno J, et al. Electrical storm in children with an implantable cardioverter defibrillator: clinical features and outcome. Heart Rhythm. 2007;4.

[5] Van der Werf C, Zwinderman AH, Wilde AA (2012). Therapeutic approach for patients with catecholaminergic polymorphic ventricular tachycardia: state of the art and future developments. Europace.

[6] Ransohoff JL (1925). Cervical sympathectomy for angina pectoris. Ann Surg.

[7] Schwartz PJ, Snebold NG, Brown AM (1976). Effects of unilateral cardiac sympathetic denervation on the ventricular fibrillation threshold. Am J Cardiol.

[8] Olde Nordkamp LRA, Driessen AHG, Odero A, et al. Left cardiac sympathetic denervation in the Netherlands for the treatment of inherited arrhythmia syndromes. Neth Heart J. 2014. doi:10.1007/s12471-014-0523-2. 10.1007/s12471-014-0523-2 PMC3954929 24522951

[9] Collura CA, Johnson JN, Moir C (2009). Left cardiac sympathetic denervation for the treatment of long QT syndrome and catecholaminergic polymorphic ventricular tachycardia using video-assisted thoracic surgery. Heart Rhythm.

[10] Hoffmann BA, Steven D, Willems S, et al. Renal Sympathetic denervation as an adjunct to catheter ablation for the treatment of ventricular electrical storm in the setting of acute myocardial infarction. J Cardiovasc Electrophysiol. 2013 Jun 12. [Epub ahead of print]. 10.1111/jce.12282 24118342

